# Remote Radio Frequency Sensing Based on 5G New Radio Positioning Reference Signals

**DOI:** 10.3390/s25020337

**Published:** 2025-01-09

**Authors:** Marcin Bednarz, Tomasz P. Zielinski

**Affiliations:** Institute of Telecommunications, AGH University of Krakow, al. Mickiewicza 30, 30-059 Krakow, Poland; mbednarz00@outlook.com

**Keywords:** integrated sensing and communication (ISAC), 5G NR, positioning reference signals (PRS), channel impulse response (CIR), remote sensing, moving vehicle detection

## Abstract

In this paper, the idea of a radar based on orthogonal frequency division multiplexing (OFDM) is applied to 5G NR Positioning Reference Signals (PRS). This study demonstrates how the estimation of the communication channel using the PRS can be applied for the identification of objects moving near the 5G NR receiver. In this context, this refers to a 5G NR base station capable of detecting a high-speed train (HST). The anatomy of a 5G NR frame as a sequence of OFDM symbols is presented, and different PRS configurations are described. It is shown that spectral analysis of time-varying channel impulse response weights, estimated with the help of PRS pilots, can be used for the detection of transmitted signal reflections from moving vehicles and the calculation of their time and frequency/Doppler shifts. Different PRS configurations with varying time and frequency reference signal densities are tested in simulations. The peak-to-noise-floor ratio (PNFR) of the calculated radar range–velocity maps (RVM) is used for quantitative comparison of PRS-based radar scenarios. Additionally, different echo signal strengths are simulated while also checking various observation window lengths (FFT lengths). This study proves the practicality of using PRS pilots in remote sensing; however, it shows that the most dense configurations do not provide notable improvements, while also demanding considerably more resources.

## 1. Introduction

Mobile networks are one of the most important innovations humans have developed. Beginning with voice and text transmission to now being one of the most advanced and popular technologies for providing wireless Internet access and communication services, it will continue to play a key role in our future. The current fifth generation of mobile networks [1,2] is constantly growing in terms of market share. Its goal is to provide the newest functionalities [3] that were not available before, one of them being integrated sensing and communications (ISAC) [4,5,6,7]. The core task of ISAC is to perform these actions at the same time using a single transmitted signal without a significant negative impact on each other. This can help save a considerable amount of energy and build large, efficient sensor networks of objects such as moving vehicles (cars, drones) in smart cities and mobile intelligent robots in modern factory automation systems.

Radar technology has a very long history [8]. In monostatic active radars, a specially designed signal is sent, and its copies coming back to the transmitter, reflected from moving targets, are then analyzed. The time delay of any reflection gives information about the distance of some target, while its frequency shift, which results from the Doppler effect, gives information about the target velocity. In order to find values of both shifts, radar typically uses the cross-ambiguity function (CAF). In CAF, samples of the received signal (RX) are multiplied by samples of the shifted-in-time and frequency transmitted signal (TX). The multiplication results are then accumulated. Co-ordinates of CAF magnitude maxima, if such peaks are present, inform us about the range and velocity of moving objects. In other words, the radar checks for which time and frequency shift values the TX signal will best correlate with the RX signal. The 2D CAF obtained in this way is called the delay-Doppler map (DDM) or range-velocity map (RVM) of the radar. In bistatic active radar systems, the first station generates and sends a radar waveform, while the second station analyzes received reflections of it. In passive radar technology [9], the publicly available signals are used for vehicle illumination. They are generated and sent by commercial digital communication transmitters, e.g., Digital Audio Broadcasting (DAB), Digital Video Broadcasting-Terrestrial (DVB-T), 4G, and 5G services.

The CAF computation, based on its definition, is very time-consuming. There are two main options: (1) first modulate the transmitted signal in frequency and then apply the fast correlation algorithm, (2) first shift the transmitted signal in time and multiply it by the received signal, then perform a spectral analysis of the result using the fast Chirp-Z transform, but only for low-frequency values. However, the computational speed-up offered by both approaches is still significantly lower than required. As a remedy to this situation, in [10], a fast proximate CAF computation method (proximate matched filter) was introduced. Here, the TX and RX signals, being compared, are divided into fragments (batches) and correlated part by part. The theoretical derivation of the method and its efficiency evaluation are presented in [11], while its comparison with other alternative approaches is given in [12]. In [13], a low-cost alternative to the CAF batch algorithm is discussed, i.e., the so-called reciprocal filtering approach. This method is, in fact, equivalent to an OFDM-based radar technique, which is characterized in the following two paragraphs.

However, looking at the fundamental problem of ISAC from the communication perspective, signal blocking is necessary in Orthogonal Frequency Division Multiplexing (OFDM)—the now widely used technique in many telecommunication services. It is a consequence of the fact that the data bits are coded in Discrete Fourier Transform (DFT) coefficients of OFDM symbols, and transmitted signal samples result from the inverse DFT of them. Additionally, each sent block of signal samples is preceded by a cyclic prefix, i.e., by the copy of the last samples. This operation guarantees that: (1) the channel impulse response does not cause interference between successive OFDM symbols, (2) linear channel convolution is visible by our communication system as a circular one, and, as a result, (3) channel equalization is straightforward: transmitted carrier states are recovered via the division of received carrier states by the DFT coefficient of the channel frequency response (CFR). The CFR is estimated using the reference carrier states of the so-called pilots, which are known to the receiver. In summary, OFDM-based digital communication systems transmit signals in blocks and have to frequently estimate the CFR using signal fragments in order to correct the received data in highly mobile, time-varying channels. Channel estimates in telecommunication receivers are calculated continuously, so extracting detailed information about reflections of transmitted signals from moving vehicles—such as their delays (vehicle distances) and Doppler shifts (vehicle velocities)—incurs little additional cost (e.g., in ISAC systems dedicated to unmanned aerial vehicles (UAVs) [14]). This is the main working principle of the so-called OFDM-based radar. Its idea is very similar to the radar CAF-by-batches methodology—see the comparison in [12]. Therefore, it is not surprising that about 15 years ago, despite the progress in radar research, an idea emerged in the communication industry to also use the communication 4G/LTE OFDM-based signals for sensing purposes, primarily with the vehicular industry in mind [15,16].

The idea and technique of OFDM-based ISAC systems are at all times of interest in 5G New Radio (NR) and in the development of behind 5G standards: 5G-A and 6G [5,6]. Ref. [17] specifies use cases for positioning services in 5G, such as accurate positioning to support augmented reality, emergency services, or UAV missions and operations, that can be simultaneously used with radar sensing or provided by it. The authors in [18] provided a complex and detailed overview of radio sensing using 5G signals, first presenting basic theories and concepts, continuing with state-of-the-art and exemplary applications, and ending with an analysis of open challenges. They also characterized the 5G OFDM-based radar. A comprehensive vision for integrated sensing and communication networks for 6G and beyond is provided in [19], reviewing signal processing, optimization, and machine learning techniques that enable ISAC networks and discussing potential applications of this technology.

When 4G or 5G OFDM waveforms are used for radar purposes, the reference pilots, exploited in them, are utilized for the CFR/CIR estimation and detection of moving objects. Currently, different 5G pilots have been investigated. In [20], the usage of 5G NR reference signals was presented for CFR/CIR-based sensing, but with reference to the example of Channel State Information Reference Signals (CSI-RS). Positioning Reference Signals (PRSs) were analyzed in [21,22]. In this context, however, in [21], RVMs were not calculated; only the velocities of the objects were estimated, whereas in [22], both range and velocity were estimated, but for just one PRS configuration. The Demodulation Reference Signal (DMRS) was also taken into account in [22,23] regarding radar detection. All three reference signals, along with the Phase Tracking Reference Signal (PT-RS) and the Tracking Reference Signal (TRS), were analyzed in [24] in the context of bistatic vehicular radar, but unfortunately, the simulation results were only provided for the CSI-RS and DMRS signals. Ref. [25] examined the feasibility of using 5G new radio reference signals for target localization in bistatic and multistatic radar configurations using time difference of arrival (TDOA) and angle of arrival (AOA) measurements of 5G NR waveforms for positioning estimation. The idea of using 5G NR reference signals for navigation was discussed in [26].

In general, the idea of joint application of pilot reference signals in the communication part of ISAC systems for channel estimation and correction, as well as in the radar part for target sensing, is one of the most important research challenges in future multi-antenna and mmWave communication [27,28,29].

The main purpose of this paper is to further investigate the feasibility of using PRS—positioning reference signals—for OFDM radar sensing in 5G mobile networks, based on the example of high-speed train (HST) detection by a 5G base station. The PRS is a highly configurable reference signal, more than others. The present paper is more communication-oriented than previous works [20,21,22,23,24,25]. Only monostatic radar is considered here: the signal is transmitted, received, and analyzed at the 5G NR base station. In contrast to other works, the HST is assumed to be a moving object—channel modeling in this case is more difficult [30]. Only the results from simulations are presented in this work; however, the simulation parameter values are based on measurements [31,32,33]. Our studies show that it is possible to build the OFDM-radar for the HST, which detects both the train range and velocity, using the PRS reference signals.

In contrast to our previous works [31,32,33], where the 5G NR waveform was used for passive coherent location (PCL) with the help of the CAF-based approach, currently, the OFDM-based active monostatic radar that exploits 5G PRS signals is tested. Before, the receiver used two antennas: a reference one (REF) looking at the 5G NR base station, and a surveillance one (SURV) looking at the region of interest. Instead of the OFDM-based radar methodology, the CAF-based one was applied: the REF signal was delayed, modulated/shifted in frequency, multiplied by the SURV signal, and integrated. In this way, the range–velocity maps (RVMs) were calculated and the vehicles detected. Alternatively, system information was recovered from the REF signal, and based on this, the 5G NR reference signals, such as PSS, SSS, SSB, and CSI-RS (without PRS), were identified and re-synthesized. They were then correlated with the SURV signal using the CAF approach. In this paper, the OFDM-based approach is applied, and PRS pilots are used.

The main innovations of this paper are as follows:

- A greater number of different 5G PRS signal configurations are tested during simulations compared to other papers on this topic;
- The HST scenario/channel is adopted from [30] for the purpose of this study;
- Complete RVMs are calculated in comparison with [21];
- The simulations parameters are set according to the values observed in our previous field experiments [31,32,33] and with respect to the 3GPP standard;
- An iterative high-resolution spectral analysis method proposed by Aboutanios and Ye [34] is used for a precise estimation of velocity;
- the findings indicate that utilizing PRS pilots in remote sensing is feasible, and the densest configurations do not offer significant enhancements while requiring substantially more resources.

The outline of this paper is as follows. In Section 2, a 5G NR grid and waveform structures are presented. Following that, in Section 2, the positioning reference signal, its advantages, and its configuration parameters are described. In Section 3, the simulation scenario and propagation channel model are defined, whereas in Section 4, the OFDM radar that uses the PRS is introduced. Finally, the simulation results are presented in Section 5. This paper ends with conclusions and proposals for future work.

## 2. 5G Resource Grid, Waveform, and Positioning Reference Signal 

**Figure 1:**
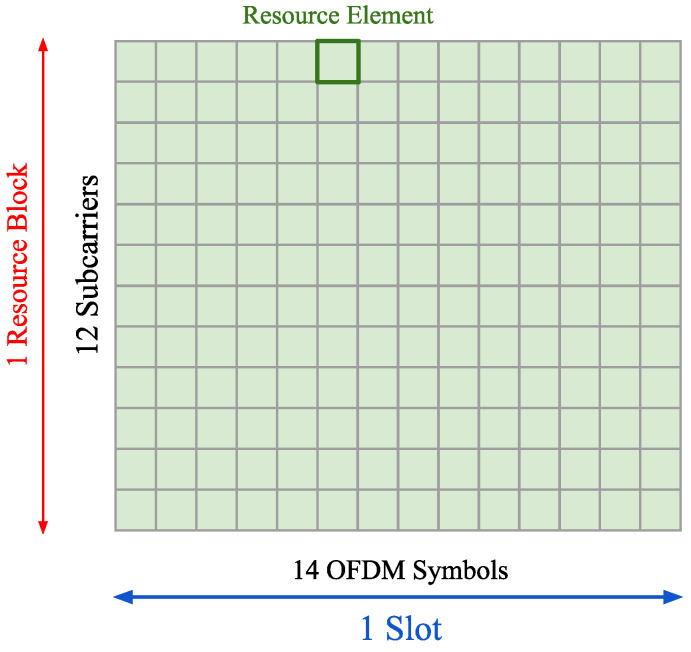
5G NR Resource Grid.

Fifth-generation mobile networks use OFDM modulation for radio communication purposes, as specified in [2]. The main idea of OFDM is to encode modulation symbols (e.g., QAM, QPSK) on orthogonal frequencies called subcarries at the same time so that they will not interfere with each other. In 5G, time is also divided into smaller pieces called symbols. Taking both subcarries and symbols, we can form a 5G grid in which we can insert data with different times and frequency resources, as illustrated in Figure 1.

The smallest element of the NR grid that consists of one subcarrier of one OFDM symbol is called the Resource Element (RE). Twelve adjacent subcarriers create a Resource Block (RB), and the RB in a specific bandwidth part is called a Physical Resource Block (PRB). Fourteen OFDM symbols bundled together form a slot, which are then combined into subframes, ten of which make up an entire 5G frame. The number of slots per subframe ($N_{Slots}^{Subframe,\mu }$) and slots per frame ($N_{Slots}^{Frame,\mu }$) varies according to 5G numerology μ as well as subcarrier spacing (SCS). There are seven numerologies in 5G NR, and their details are presented in Table 1. The whole 5G frame always has a duration time of 10 ms.

The PRS pilots, which are used for radar sensing, are inserted into the RE of the 5G NR grid, together with user data. Therefore, the more pilots are used, the fewer data are transmitted; that is, the available communication bit stream is limited. For this reason, it is important to find a pilot configuration that allows for precise radar detection but does not limit transmission throughput too much.After pilot placement, the OFDM modulation is performed as follows: each OFDM symbol is converted to the time domain using the $N_{fft}$-point inverse fast Fourier transform (IFFT), after which the $N_{cp}$ last samples of the symbol are copied to its beginning. This operation is called a cyclic prefix (CP) insertion. The CP length depends on the symbol number in a given subframe. The lengths of $N_{fft}$ and $N_{cp}$ are defined in Section 5.3 of [2]. Once the modulation is performed on all OFDM symbols, the time domain waveform is transmitted through the radio channel. On the receiver side, the cyclic prefix is removed, the $N_{fft}$-point Fast Fourier Transform (FFT) is performed, and the symbol is assigned to the 5G resource grid.

For positioning tasks, the PRS is used as a pilot. One may wonder why pilots are necessary for radar sensing since the 5G NR base station knows exactly what it has sent through the radio channel in the downlink direction and can perform sensing on this basis. The answer is that the traffic on downlink channels is highly dependent on the current network conditions and the number of connected users, which implies high variability and sometimes (e.g., during night hours) a lack of downlink traffic at all. Therefore, the empty resource elements have to be interpolated, which might be time consuming depending on the number of empty REs. Pilots appropriately configured for this purpose are regular and deterministic, which allows us to skip the interpolation procedure. PRS is defined in 3GPP TS 38.211 [2] in Section 7.4.1.7 and in 3GPP TS 38.214 [35] in Section 5.1.6.5. PRS is composed from Gold sequences. This implies that the PRS sequence has good (high) auto-correlation characteristics and very low cross-correlation with sequences of other reference signals and data. There are many parameters that determine how PRS can be inserted into the 5G NR resource grid, making it more flexible than other reference signals and allowing for different occupation densities to be defined in the time/frequency domain. Moreover, the standard provides the possibility of configuration of more than one PRS resource. This feature is helpful in designing the PRS configuration for an OFDM-based radar and allows for beam-sweeping. The collection of multiple PRS resources is called the PRS resource set. The 3GPP standard also allows for a muting-pattern, which can be used to avoid PRS transmission in certain situations. This helps prevent interference from neighboring cells. The most important parameters that configure the PRS mapping to physical resources and time slots are listed in Table 2.

**Figure 2:**
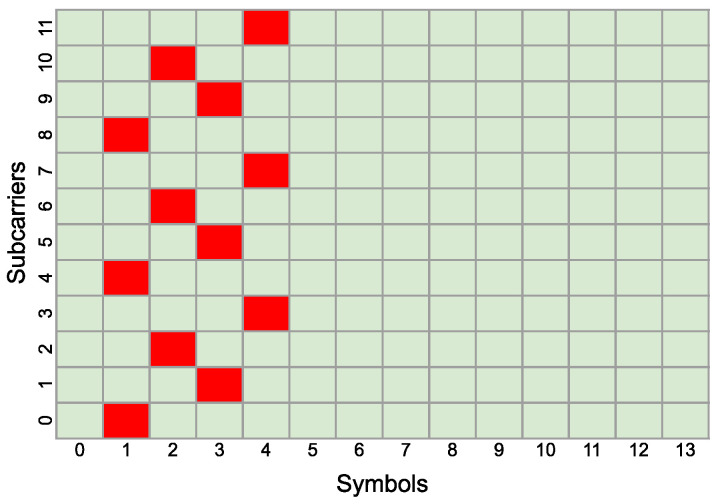
Physical resource mapping of PRS with $K_{comb}^{PRS}$ = 4, $L_{PRS}$ = 4, and $k_{offset}^{PRS}$ = 1. The PRS resource is marked with red color.

**Figure 3:**
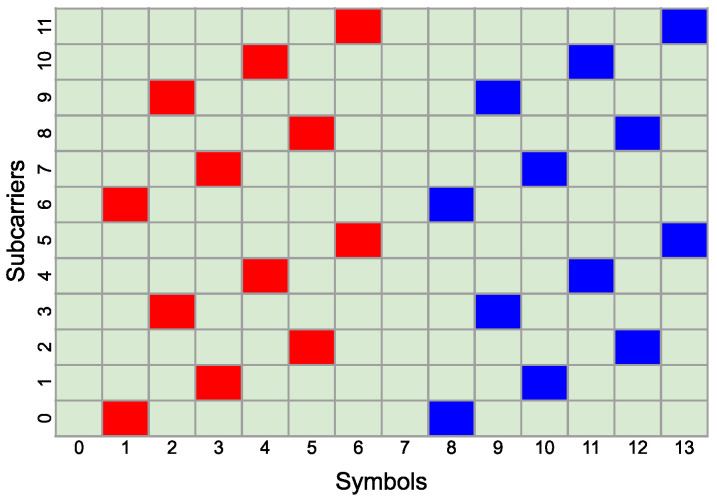
Physical resource mapping of two PRS resources with $K_{comb}^{PRS}$ = 6, $L_{PRS}$ = 6 and $k_{offset}^{PRS}$ = 1, and $k_{offset}^{PRS}$ = 8. The first PRS resource is marked with the red color, and the second PRS resource is marked with the blue color.

For visualization purposes, in Figure 2 and Figure 3, two exemplary PRS configurations are presented, which will be used later for sensing services. The first configuration consists of one PRS resource, for which $K_{comb}^{PRS}$ is equal to 4. This implies that the PRS pilot will be filled in every fourth sub-carrier in the single symbol. $L_{PRS}$ is set to 4; therefore, the PRS resource will occupy 4 symbols in a single slot, and $l_{start}^{PRS}$ is set to 1, so the PRS resource will occupy symbols from 1 to 4. Figure 2 represents this PRS configuration in the context of 1 slot and 12 subcarriers. Despite the fact that the 0-th and $7*2^{\mu }$-th [2] symbol in the resource block can have a long cyclic prefix, instead of the short one, the PRS pilots will be inserted in equal time intervals on one subcarrier, i.e., once per one carrier in the 12-symbol resource block.

The second configuration, shown in Figure 3, consists of two PRS resources, both with $K_{comb}^{PRS}$ = 6 and $L_{PRS}$ = 6. Since we have two PRS resources in this set, the $l_{start}^{PRS}$ for the first is set to 1 and the $l_{start}^{PRS}$ for the second resource is set to 8. This means that PRS pilots will be inserted in approximately equal time intervals on one subcarrier. This is only approximate because the 0-th and the $7*2^{\mu }$-th [2] symbol can have a long, rather than short, cyclic prefix. However, as will be shown in simulations, even such slightly irregular CFR sampling is sufficient to measure the Doppler effect via FFT.

**Figure 4:**
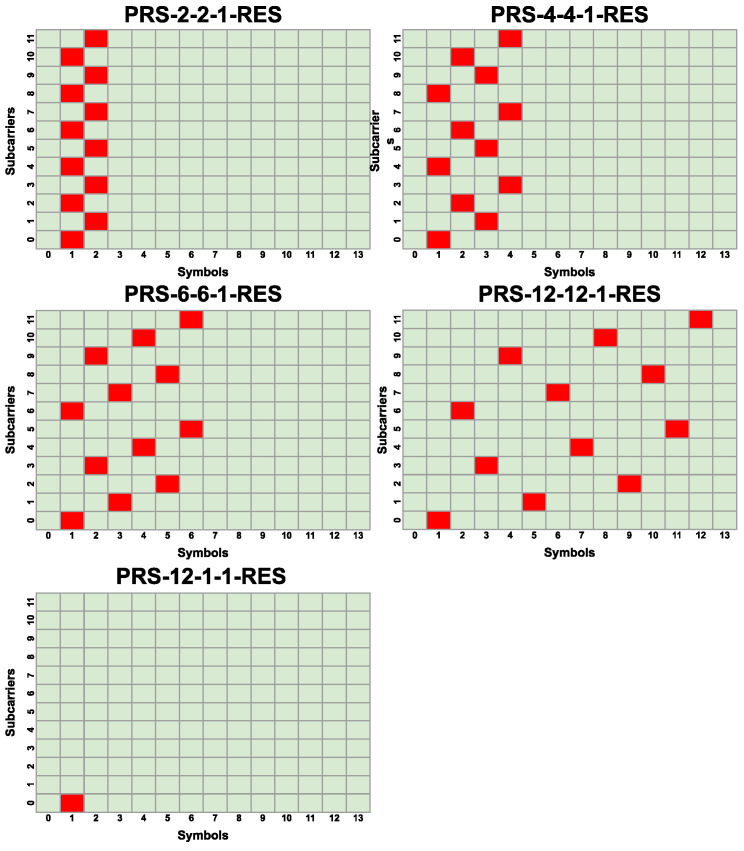
Different PRS configurations with one PRS resource. The PRS resource is marked with red color.

**Figure 5:**
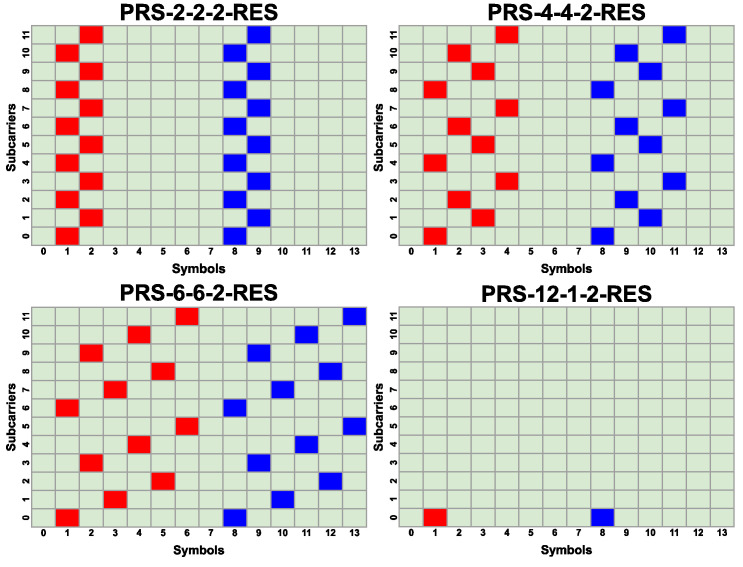
Different PRS configurations with two PRS resources. The first PRS resource is marked with the red color, and the second PRS resource is marked with the blue color.

Different PRS configurations are shown in Figure 4 and Figure 5. Their denotation PRS-X-Y-Z-RES uses the following convention: X = $K_{comb}^{PRS}$, Y = $L_{PRS}$, and Z is the number of PRS resources used in the configuration.

## 3. Investigated Radar Scenario and Propagation Channel Model

**Figure 6:**
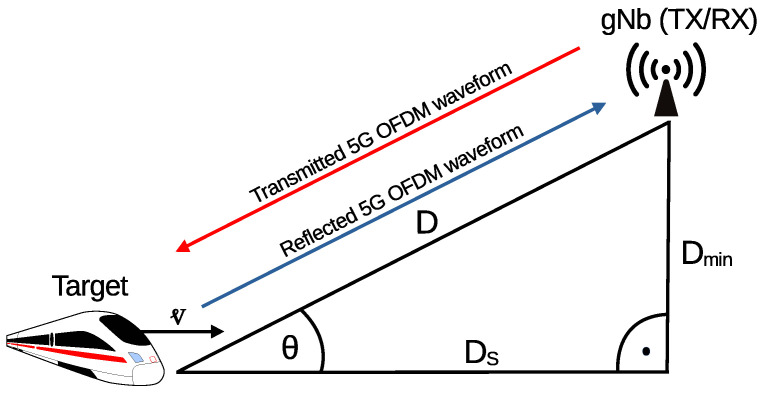
Study scenario with one target.

The scenario investigated in this study is presented in Figure 6 and is the same as the one provided in 3GPP TS 38.101-4 Annex B.3 [30], which is the high-speed train scenario. A train is inherently an elongated target for tracking, which negatively affects the quality of detection. In this paper, this effect is simplified by considering it as a point target, i.e., assuming that it is moving exactly in the direction to/from (toward/against) the base station (gNb). We consider only the monostatic radar, which means that gNb uses transmitted signal $x_{TX}\left(t\right)$ and received signal $y_{RX}\left(t\right)$ to identify reflections of $x_{TX}\left(t\right)$, which are present in $y_{RX}\left(t\right)$. The simulation scenario consists of one gNb station that is mounted at $D_{min}$ meters above ground and one target that is $D_{s}$ meters from it in a straight line and ideally moving toward or against it with constant velocity vm/s. Therefore, the distance *D* can be expressed as follows:

(1) $$D=\sqrt{D_{s}^{2}+D_{min}^{2}}.$$

The two-way propagation time within the distance *D* can be expressed as follows:

(2) $$\tau =\frac{2D}{c},$$

where *c* denotes the speed of light. As per 3GPP TS 38.101-4 Annex B.3 [30], the Doppler frequency shift is dependent on cos(θ), which can vary in time because the target moves toward/against the base station and can be expressed as follows:

(3) $$\cos\left(\theta \left(t\right)\right)=\frac{{\left(D_{s}-vt\right)}^{2}}{\sqrt{{\left(D_{s}-vt\right)}^{2}+D_{min}^{2}}}.$$

The maximum Doppler shift is obtained when the target and gNb are positioned at the same height, and it is equal to the following:

(4) $$f_{max}^{\left(D\right)}=\frac{2v}{\lambda },\;\lambda =\frac{c}{f_{c}},$$

where $f_{c}$ is a carrier frequency of the waveform. Thus, the Doppler shift in a specific time *t* is given as follows:

(5) $$f^{\left(D\right)}\left(t\right)=f_{max}^{\left(D\right)}\cos\left(\theta \left(t\right)\right).$$

Additionally, the delay of the transmitted signal $x_{TX}\left(t\right)$ by τ (2) causes a linear phase shift of the signal Fourier spectrum X(f):

(6) $$x_{TX}(t-\tau )↔X\left(f\right)e^{-j2\pi f\tau }.$$

Finally, after using (5), a single reflected signal $y_{p}\left(t\right)$ from the path *p* with complex-value reflection gain $g_{p}$ (the strength of the reflection), Doppler frequency shift $f_{p}^{\left(D\right)}$, and delay $\tau_{p}$ is defined in a specific time *t* as follows:

(7) $$y_{p}\left(t\right)=g_{p}x_{TX}\left(t-\tau_{p}\right)e^{j2\pi f_{p}^{\left(D\right)}\left(t\right)t}.$$

After the following substitution:

(8) $$h_{p}\left(t\right)=g_{p}e^{j2\pi f_{p}^{\left(D\right)}\left(t\right)t},$$

(7) can be rewritten as:

(9) $$y_{p}\left(t\right)=h_{p}\left(t\right)x_{TX}\left(t-\tau_{p}\right),$$

The signal $y_{RX}\left(t\right)$ in a radar receiver is, in general, a sum of reflections of the following form (9):

(10) $$y_{RX}\left(t\right)=\sum_{p=1}^{P}y_{p}\left(t\right).$$

The weight $h_{p}\left(t\right)$ of the complex value channel, associated with the path *p*-th, has magnitude $|g_{p}|$ and oscillates with drifting frequency $f_{p}^{\left(D\right)}\left(t\right)$ (5), which has to be estimated in a radar sub-section of the receiver. In the investigated HST scenario, we assume only two reflections: one strong reflection p=1 from the motionless object, treated as a disturbing clutter (for simplicity, it is a transmitted signal itself), and one weak reflection from the moving train:

(11) $$\begin{array}{cc} y_{RX}\left(t\right) & =y_{1}\left(t\right)+y_{2}\left(t\right)\\ \; & =x_{TX}\left(t\right)+h_{2}\left(t\right)x_{TX}\left(t-\tau_{2}\right)\\ \; & =x_{TX}\left(t\right)+g_{2}e^{j2\pi f_{2}^{\left(D\right)}\left(t\right)t}x_{TX}\left(t-\tau_{2}\right). \end{array}$$

## 4. OFDM Radar Sensing Method

### 4.1. Monostatic OFDM Radar Sensing

**Figure 7:**
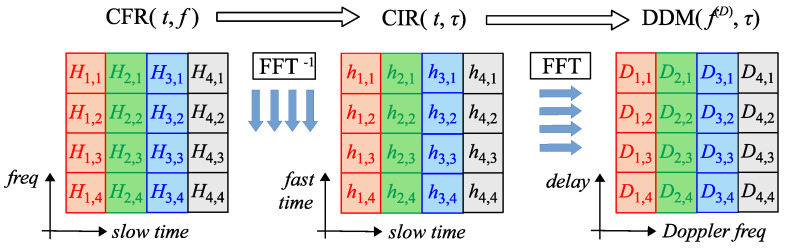
Principle of the OFDM-based radar exploiting PRS.

The principle of the OFDM-based radar is explained in Figure 7. Let us define $RG_{TX}$ as the transmitted resource grid and $RG_{RX}$ as the received resource grid, obtained via OFDM demodulation of the received signal. Therefore, estimation of the channel frequency response $H(k_{p},l_{p})$ can be obtained as follows:

(12) $$H\left(k_{p},l_{p}\right)=\frac{RG_{RX}\left(k_{p},l_{p}\right)}{RG_{TX}\left(k_{p},l_{p}\right)},$$

where $k_{p}$ denotes the *k*-th subcarrier used by the PRS pilot, $l_{p}$ denotes the *l*-th symbol used by the PRS pilot, and the pair $\left(k_{p},l_{p}\right)$ denotes the particular resource element in the resource grid. For the sensing task, all resource elements of H(k,l) that do not contain PRS pilots are zeroed. Then, the channel impulse response (CIR) is calculated from the already estimated channel frequency response (CFR) by performing an inverse FFT on all spectra of OFDM symbols that contain PRS pilots. Next, the Doppler frequency shifts are found by performing an FFT upon time-varying CIR weights, separately, tap by tap. In this way, the resultant delay–Doppler (range–velocity) matrix (DDM/RVM) is obtained.

**Figure 8:**
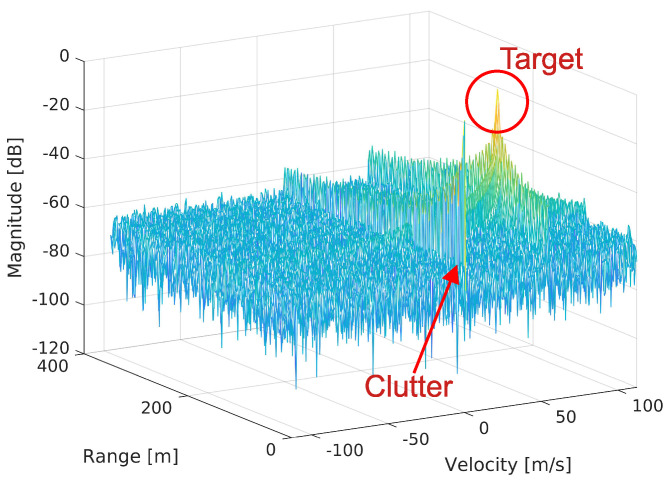
Exemplary range−velocity matrix (RVM) with one target. The warmer the color, the greater strength of the reflection.

Since CIR cannot be longer than a longer cyclic prefix (CP), the CIR matrix can be limited to the $N_{cp}^{long}$ or $N_{pilots}^{symbol}$ first rows, depending on which is smaller, where $N_{cp}^{long}$ denotes the size of the longer 5G CP and $N_{pilots}^{symbol}$ denotes the number of PRS pilots in one OFDM symbol. Let $N_{pilots}^{sc}$ be the number of all PRS pilots placed on a subcarrier. Since the FFT spectrum of any CIR weight is periodic (i.e., repeated by $N_{pilots}^{sc}$ columns due to repeating blocks of zeros in the CFR matrix of the PRS pilots), we should consider only the first $N_{pilots}^{sc}$ columns of the DDM matrix. By performing a circular shift of the columns of the resulting DDM matrix by $\frac{N_{pilots}^{sc}}{2}+1$ elements to the right, the 0Hz Doppler frequency shift is placed in the center of the DDM matrix. The delay–Doppler matrix can be easily transformed to the range–velocity matrix by converting delay to distance and Doppler shift to velocity (by applying Equations (16) and (20) to the row and column indices, respectively). An exemplary range–velocity matrix that was obtained after all the operations described above is presented in Figure 8. In the center of this figure, a strong clutter component is seen, originating from motionless objects in (10). In this study, $x_{TX}\left(t\right)$ plays the role of a disturber (see (11)). On the right side of the clutter, we see the peak originating from a moving object. It is significantly higher than in field measurements [31,32,33] and in the performed simulations in order to show its spectral side-lobes better and to visualize a risk of masking a weak echo with a strong one. In this study, we consider scenarios with only one moving object at the same time, i.e., the HST. The influence of motionless clutter can be reduced by any clutter removal algorithm (for example, by the extensive cancellation algorithm ECA-PLUS [36]); however, this topic is not addressed in this study.

### 4.2. Range Estimation

The maximum achievable sensing range $r_{max}$ is equal to the following:

(13) $$r_{max}=\frac{(N_{limit}^{range}-1)\cdot c\cdot dt}{2},$$

where $N_{limit}^{range}$ is defined as follows:

(14) $$N_{limit}^{range}=min\left(N_{cp}^{long},N_{pilots}^{symbol}\right),$$

and $N_{cp}^{long}$ denotes the length of the 5G NR longer cyclic prefix, $N_{pilots}^{symbol}$ denotes the number of PRS pilots in one OFDM symbol, and dt is the sampling period (inverse of the sampling frequency: $\frac{1}{f_{s}}$). The resolution of range estimation $r_{res}$ is given by the following formula:

(15) $$r_{res}=\frac{r_{max}}{\left(N_{limit}^{range}-1\right)}=\frac{c\cdot dt}{2}.$$

Let us define $ind_{r}$ as the index of the CIR row with a maximum value. Then, the estimated range can be expressed as follows:

(16) $$r_{est}=\frac{ind_{r}\cdot c\cdot dt}{2}.$$

### 4.3. Velocity Estimation

The maximum observable Doppler frequency shift $f_{max}^{\left(D\right)}$ is given by the following formula:

(17) $$f_{max}^{\left(D\right)}=\frac{f_{s}^{\left(p\right)}}{2}-\frac{f_{s}^{\left(p\right)}}{N_{pilots}^{sc}}=\frac{f_{s}^{\left(p\right)}}{2}-\frac{1}{T},$$

where $f_{s}^{\left(p\right)}$ denotes the sampling frequency of the channel frequency response by PRS pilots in time/symbol axis, which is equal to $\frac{N_{pilots}^{sc}}{T}$, while *T* denotes the time duration of the transmitted/received signal fragment. Therefore, the Doppler frequency shift resolution $f_{res}^{\left(D\right)}$ can be expressed as follows:

(18) $$f_{res}^{\left(D\right)}=\frac{f_{s}^{\left(p\right)}}{N_{pilots}^{sc}}=\frac{1}{T}.$$

Let us define $idx_{f}^{\left(D\right)}$ as the index of the CIR column with the maximum value. The estimated Doppler frequency shift $f_{est}^{\left(D\right)}$ is given as follows:

(19) $$f_{est}^{\left(D\right)}=\left[ind_{f}^{\left(D\right)}-\left(\frac{N_{pilots}^{sc}}{2}+1\right)\right]\cdot f_{res}^{\left(D\right)}.$$

Then, the estimated velocity $v_{est}$ can be expressed as follows:

(20) $$v_{est}=\frac{f_{est}^{\left(D\right)}\cdot c}{2f_{c}}.$$

## 5. Results

### 5.1. Simulation Setup

Simulations were performed using MATLAB R2023B and 5G Toolbox. The Frequency Division Duplex (FDD) mode was exploited. The assumed downlink carrier parameters are presented in Table 3. The values of the radar target parameters used in the simulations are given in Table 4, i.e., range, velocity, and reflection gain. The first two parameters do not require additional explanation. Reflection gain defines how strong the echo signal is in the signal recorded by the receiver (see Equation (11)). For $g=\frac{0.01*(1+1j)}{\sqrt{2}}$, the echo component is 100 times weaker than the light-of-sight component. This reflection gain value was selected based on our previous field studies [31,32,33].

Table 5 shows the PRS configuration parameters shared across all configurations. The detailed parameters of each PRS configuration used in the simulation are presented in Table 6, which has columns indicating the number of REs occupied by PRS in a single slot and the total overhead caused by that. The more REs are intended for PRS, the larger the overhead, which means that fewer REs can be dedicated to communication tasks. This can result in lower bit throughput.

PRS configurations are primarily categorized into two groups: those with two PRS resources (PRS-6-6-2-RES, PRS-4-4-2-RES, PRS-2-2-2-RES), and those with one PRS resource (PRS-2-2-1-RES, PRS-4-4-1-RES, PRS-6-6-1-RES, PRS-12-12-1-RES). The first group has the denser pilot array, utilizing two times more pilots compared to each configuration of the second group. Such an arrangement should enhance the sensing capabilities in poor radio conditions (e.g., lower SNR). The use of two PRS resources enables the placement of two pilots on the same subcarrier. This increases the maximum observable Doppler frequency shift (see Equation (17)) but at the same time increases the pilot overhead. The second group of configurations with only one PRS resource exhibits a lower maximum observable Doppler frequency shift and decreased sensing capabilities; however, it is beneficial when additional resources are required to obtain higher bit throughput in the communication part of the system. Two of the configurations presented (PRS-12-1-2-RES and PRS-12-1-1-RES) are not currently supported by the 3GPP standard due to the invalid value of $L_{PRS}$, but they are included in the simulations to show their potential benefits for 5G networks to have such configurations available. These configurations occupy very few REs in a single slot, which allows the operator to allocate more resources for data transmission. Moreover, the usage of these two extra configurations would allow better adjustment to radio channel conditions.

The selection of values of the target parameters follows the rules described in Section 3, and only one target is present at the same time in a single iteration of the simulation. The radar targets were selected in such a way that neither velocity nor range exactly fell into the IFFT/FFT spacing (bins) of the CIR and DDM matrices (i.e., fractional, not integer, position of targets in DDMs was assumed). For this reason, blur occurs in the DDM. This is the worst-case scenario that can occur in field conditions; therefore, it is used to measure the performance quality of the different PRS configurations used in simulations. For velocity estimation, the high-resolution spectral analysis method of Aboutanios and Ye was used [34].

**Table 3:** Carrier parameters for performed simulations.

Parameter	Value	Meaning
μ	1	5G numerology
RB	133	Number of resource blocks
BW	50	Bandwidth [MHz]
Nfft	2048	Number of FFT points for OFDM de/modulation
$N_{cp1}$	176 (7.9%)	Longer cyclic prefix (% of the whole OFDM symbol size)
$N_{cp2}$	144 (6.5%)	Shorter cyclic prefix (% of the whole OFDM symbol size)
$f_{c}$	2.655	Carrier frequency [GHz]
$f_{s}$	61.44	Sample rate [MHz]

**Table 4:** Radar targets used in simulations.

Parameter	Values
Range [m]	54.67, 213.42, 123.10, 245.140
Velocity m/s	16.18, 32.53, −9.91, −43.28
Reflection gain	$\frac{0.01*(1+1j)}{\sqrt{2}}$

**Table 5:** PRS configuration parameters shared among all configurations in Table 6.

Parameter Name	$k_{\mathrm{offset}}^{\mathrm{PRS}}$	$N^{\mathrm{RB}}$	$I_{\mathrm{start}}^{\mathrm{PRB}}$	$T_{\mathrm{per}}^{\mathrm{PRS}}$	$T_{\mathrm{offset}}^{\mathrm{PRS}}$	$T_{\mathrm{offset},\mathrm{res}}^{\mathrm{PRS}}$	$T_{\mathrm{rep}}^{\mathrm{PRS}}$	$T_{\mathrm{gap}}^{\mathrm{PRS}}$
Value	0	104	0	32	0	0	32	1

**Table 6:** PRS configurations for simulations.

Short Name	$K_{comb}^{PRS}$	$L_{PRS}$	$l_{start}^{PRS}$	No. PRS Resources	$p_{f_{s}}$ [Hz] for μ=1	Occupied RE in Single Slot with 12 Subcarriers	Overhead [%]
PRS-2-2-1-RES	2	2	1	1	2000	12	7.1
PRS-4-4-1-RES	4	4	1	1	2000	12	7.1
PRS-6-6-1-RES	6	6	1	1	2000	12	7.1
PRS-12-12-1-RES	12	12	1	1	2000	12	7.1
PRS-12-1-1-RES	12	1	1	1	2000	1	0.6
PRS-6-6-2-RES	6	6	1, 8	2	4000	24	14.2
PRS-4-4-2-RES	4	4	1, 8	2	4000	24	14.2
PRS-2-2-2-RES	2	2	1, 8	2	4000	24	14.2
PRS-12-1-2-RES	12	1	1, 8	2	4000	2	1.2

### 5.2. Signal-to-Noise Ratio Impact on Radar Sensing

In this set of simulations, the impact of the signal-to-noise ratio (SNR) on radar detection is measured. For this purpose, the additive white Gaussian noise (AWGN) is used to simulate different levels of SNR. Noise is added directly to the radio signal $y_{RX}$, which is defined by Equation (11). All targets specified in Table 4 are used, and the observation time is 100 ms (ten 5G frames).

#### 5.2.1. Peak-to-Noise-Floor Ratio

The peak-to-noise-floor ratio (PNFR) is defined as follows [37]:

(21) $$PNFR=20\log_{10}\left(\frac{A_{reflection}}{A_{noise}}\right),$$

where $A_{reflection}$ (in dB) denotes the peak magnitude of the radio signal reflected by the moving target in the RVM matrix, and $A_{noise}$ (in dB) denotes an estimated average noise floor magnitude of the RVM matrix. The average noise floor magnitude is estimated by taking a small square (45 × 45 points of RVM) where only noise appears and calculating its average value.

**Figure 9:**
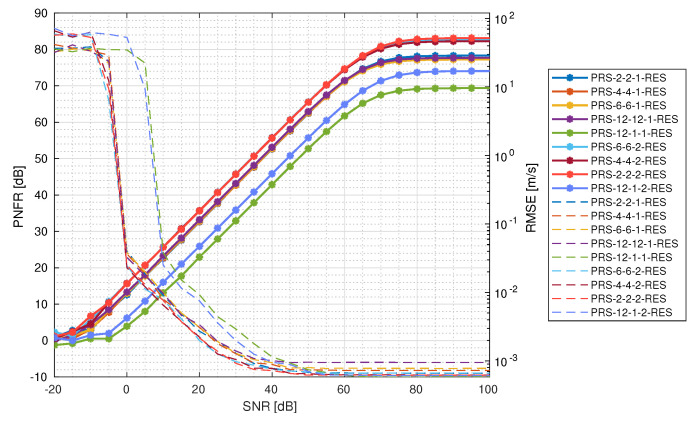
PNFR for different PRS configurations (solid lines with asterisk markers) together with velocity RMSE as a reference (dashed lines).

In Figure 9, PNFR is presented together with the root mean square error (RMSE) of the velocity estimation based on the Aboutanios and Ye method [34], as a reference. It can be seen that the PNFR becomes constant at 80 dB of the input SNR. Higher values do not result in a better PNFR; moreover, the RMSE velocity becomes constant at about 50 dB of the input SNR, so it is clear that the increase in PNFR is not reflected in a more precise velocity measurement. PRS-2-2-2-RES, PRS-4-4-2-RES, and PRS-6-6-2-RES are the configurations with the best PNFR performance, reaching about 83 dB. These configurations also have the highest PNFR for all levels of SNR. The next best are PRS-12-12-1-RES, PRS-6-6-1-RES, PRS-4-4-1-RES, and PRS-2-2-1-RES, which reach a maximum PNFR of approximately 77 dB. The two worst-performing configurations are PRS-12-1-2-RES and PRS-12-1-1-RES. They stand out from the rest, with the first one achieving a maximum PNFR of 74 dB, and the second one achieving only 70 dB. According to the velocity estimation, all configurations, apart from PRS-12-1-2-RES and PRS-12-1-1-RES, achieve similar performance for a wide range of SNRs. All of them achieve RMSE values under $10^{-3}$ m/s, with the two worst configurations reaching this later than the others. In general, we see only a slight difference between configurations with one and two PRS resources. Allocating two times more resource elements for PRS does not guarantee sufficient gain in terms of PNFR. More detailed analysis of velocity estimation is presented in the next subsection.

#### 5.2.2. Root Mean Square Error of Velocity Estimation

**Figure 10:**
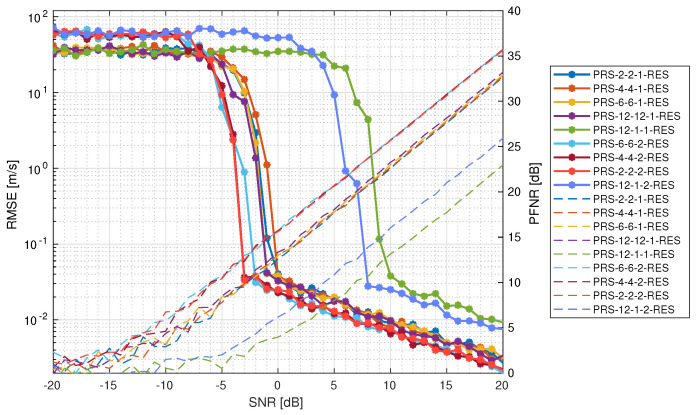
Root mean square error of velocity estimation for different PRS configurations (solid lines with asterisk markers) together with PNFR as a reference (dashed lines).

Figure 10 presents the RMSE of the velocity estimation. It is apparent that PRS configurations with two PRS resources achieve satisfactory RMSE values (under $10^{-1}$ m/s) earlier than others. This happens because they occupy more REs in the same slot. The best results are obtained by the PRS-2-2-2-RES, PRS-4-4-2-RES, and PRS-6-6-2-RES configurations, where the first two achieved satisfactory RMSE values at −3 dB, and the last achieved it at −2 dB of the input SNR. It was obtained together with PNFR at the level of ∼12.5 dB and ∼13 dB, respectively. The next four best are the configurations that had 1 PRS resource configured. PRS-12-12-1-RES achieved a satisfactory RMSE at −1 dB in conjunction with PNFR at ∼12 dB, along with PRS-6-6-1-RES. PRS-2-2-1-RES and PRS-4-4-1-RES were also very close to each other, reaching a satisfactory RMSE at 0 dB of the input SNR with simultaneous ∼13 dB of the PNFR. The worst-performing configurations were those with only two REs allocated for PRS per slot (PRS-12-1-2-RES) and one RE allocated for PRS per slot (PRS-12-1-1-RES). The fact that they occupied the least amount of REs allowed us to allocate more resources for the communications tasks; nevertheless, this implies that they require the best channel conditions for successful radar sensing. The configuration with two PRS resources achieved a satisfactory RMSE at 8 dB of the input SNR with simultaneous ∼13.5 dB of PNFR and a PRS-12-1-1-RES configuration with 1 resource at 10 dB of the input SNR with simultaneous PNFR at the level of ∼13 dB. Overall, the configurations with 1 and 2 PRS resources were close to each other in terms of performance. The difference in SNR when achieving a satisfactory RMSE was only 2 dB, while two PRS resources occupied twice more resources in the 5G grid. Considering this, configurations with one PRS resource are much better in terms of performance/overhead ratio.

#### 5.2.3. Root Mean Square Error of Range Estimation

**Figure 11:**
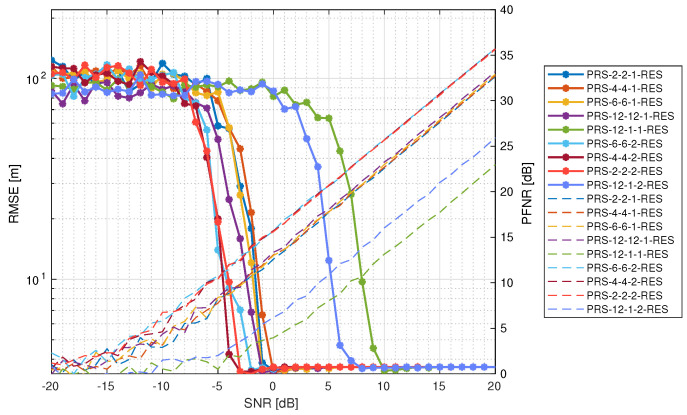
Root mean square error of range estimation for different PRS configurations (solid lines with asterisk markers) together with PNFR as a reference (dashed lines).

The range estimation RMSE is presented in Figure 11. The best-performing configurations are PRS-4-4-2-RES, PRS-2-2-2-RES, and PRS-6-6-2-RES, where the first two reach the minimum range estimation RMSE at −3 dB of the input SNR with simultaneous PNFR equal to ∼12.5 dB, and the last achieves it at −2 dB with PNFR at the level of ∼13 dB. It is noticeable that PRS-12-12-1-RES is the next-best configuration, together with PRS-6-6-1-RES and PRS-2-2-1-RES. Although the first configuration spans 12 symbols in a single slot, it has pilots only on every 12-th subcarrier. Despite that, all of them reached a minimal RMSE at−1 dB of the input SNR with simultaneous PNFR at a level of ∼12 dB. It should be mentioned that PRS-12-12-1-RES converged faster to its minimal RMSE than PRS-6-6-1-RES and PRS-2-2-1-RES. PRS-4-4-1-RES reached a minimal RMSE at 0 dB of the SNR and with PNFR at ∼13 dB. Similarly, in the case of velocity estimation, PRS-12-1-2-RES and PRS-12-1-1-RES perform the worst, with the first configuration reaching a minimal RMSE at 8 dB of SNR with PNFR at ∼13.5 dB and the second at 10 dB of SNR with PNFR at ∼13 dB.

### 5.3. Reflection Gain Impact on PNFR Level

In this section, the impact of reflection gain on the peak-to-noise floor ratio is measured. For this purpose, a radar target from Table 4 was chosen (with a velocity of 32.53 m/s and a range of 213.42 [m]), and simulations were performed with three different levels of reflection gain: $\frac{0.01*(1+1j)}{\sqrt{2}}$, $\frac{0.005*(1+1j)}{\sqrt{2}}$, and $\frac{0.001*(1+1j)}{\sqrt{2}}$. The legend of the figures is supplemented with the suffix G = X, where X denotes the amplitude of the reflection gain for which the PNFR was measured.

#### 5.3.1. Reflection Gain Impact on Function of SNR Level

**Figure 12:**
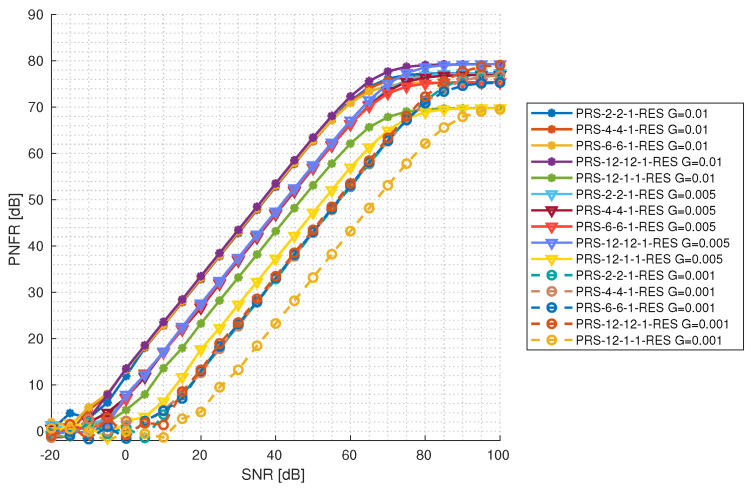
PNFR for different PRS configurations with 1 PRS resource as a function of SNR for different levels of reflection gains.

**Figure 13:**
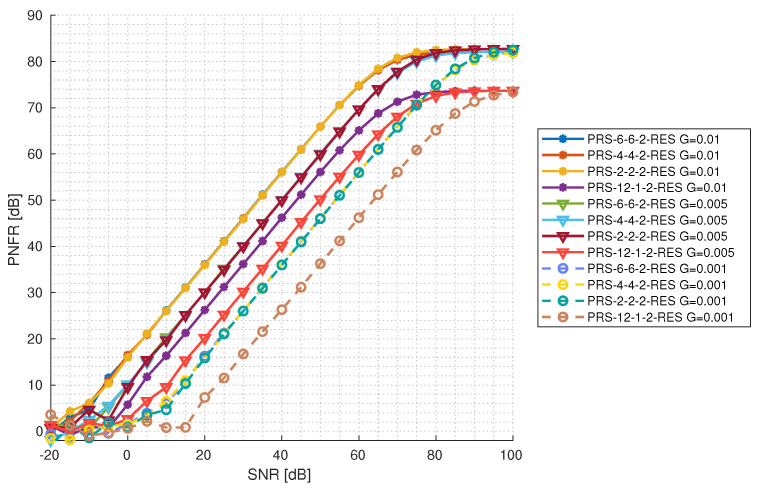
PNFR for different PRS configurations with 2 PRS resources as a function of SNR for different levels of reflection gains.

In Figure 12 and Figure 13, the PNFR level depending on the reflection gain and the SNR level is presented for configurations with one and two PRS resources, respectively. For this purpose, the observation time of ten 5G frames (100 ms) was chosen. It can be seen that configurations with the same reflection gain increase their PNFR at the same rate, exactly as in the simulation before. They also achieve a maximum PNFR approximately at the same level; in the case of configurations with one PRS resource, the maximum PNFR is 80 dB and the lowest one is 75 dB. It is worth mentioning that PRS-12-1-1-RES is underperforming compared to other configurations with the same reflection gain. It requires a higher SNR to start to increase the PNFR, and it achieves a maximum PNFR at a level of 70 dB. The same situation occurs in the case of configurations with two PRS resources. All configurations, except for PRS-12-1-2-RES, gain in their PNFR at the same rate (considering configurations with the same reflection gain) and achieve a maximum PNFR at approximately the same level of 82 dB. PRS-12-1-2-RES underperforms and requires a higher SNR level to start increasing in PNFR for all reflection gains and achieves a maximum PNFR at a level of 74 dB. In general, lower gain values require higher SNR values to start increasing in PNFR, yet at the end, they achieve a maximum PNFR at a level similar to the others. This suggests that detecting targets with very low reflection gains is feasible under excellent radio conditions. Furthermore, optimizing the PRS configuration according to radio conditions can enhance this capability.

#### 5.3.2. Reflection Gain Impact as a Function of 5G Frame Number

**Figure 14:**
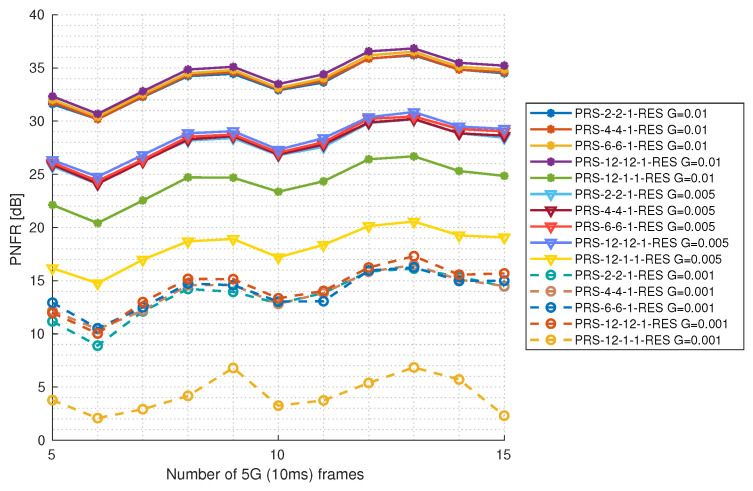
PNFR for different PRS configurations with 1 PRS resource as a function of the number of frames taken for observation for different levels of reflection gains.

**Figure 15:**
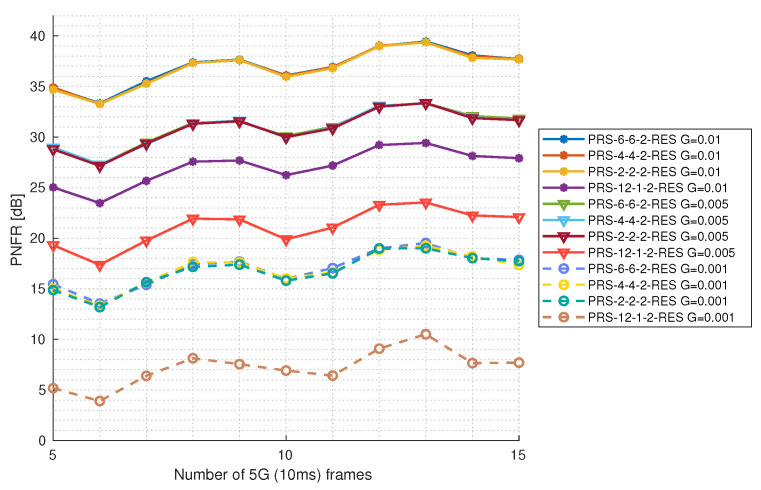
PNFR for different PRS configurations with 2 PRS resources as a function of the number of 5G frames taken for observation for different levels of reflection gains.

In Figure 14 and Figure 15, the PNFR level is presented depending on the reflection gain and the number of observed 5G frames (number of frames taken into account while calculating CIR and RVM matrices) for configurations with one and two PRS resources, respectively. For this purpose, the constant level of SNR at 20 dB was used. The maximum number of 5G frames is 15. This is because [2] explicitly states that PRS pilots cannot be placed on the same symbols as the Synchronization Signal Block with Physical Broadcast Channel (SS/PBCH). SS/PBCH is transmitted periodically, with a periodicity ranging from 5 ms to 160 ms. To simplify, we assume SS/PBCH periodicity of 160 ms and exclude the frame on which SS/PBCH occurs from sensing. This gives us a maximum of 150 ms of observation time (fifteen 5G frames). It can be seen that different PRS configurations with the same number of PRS resources perform very similar at the same reflection gain level. Interestingly, the PNFR does not increase linearly, as before in the function of the SNR level, but it has some local maximums; for example, at thirteen 5G frames. This is because for the different numbers of 5G frames that were used to calculate the CIR and RVM, we will obtain different sizes of these matrices. This implies that for different numbers of 5G frames, velocity sensing will be performed with a different resolution (see Equation (18)). As said above in Section 5, the radar targets are designed in such a way that for ten 5G frames of observation time, neither velocity nor range falls exactly into the bins of the CIR and RVM matrices, but right between the two adjacent bins. The spectrum spreads on them, resulting in a lower magnitude and therefore lower PNFR. As as result, for the observation time of ten 5G frames, we have a local minimum, and PNFR appears worse than on the other numbers of 5G frames. This example is very good at showing how important the sensing resolution is to radar performance and what happens when our target is ideally between two bins of the CIR matrix. Ten 5G frames of observation time lose about 4 dB of PNFR to the thirteen 5G frames of observation time. However, increasing the number of 5G frames is generally associated with a higher PNFR, with the gain reaching up to 3 dB when considering values at five and fifteen 5G frames. PRS-12-1-1-RES and PRS-12-1-2-RES stand out from the rest of the configurations: the first one seems to have a problem with the lowest gain because the PNFR does not improve at all. For these configurations, better radio conditions are needed to detect targets with such a low reflection gain.

## 6. Conclusions

In this paper, we have presented new simulation results from the use of a 5G PRS-based OFDM radar for the detection of high-speed trains. Many PRS configurations have been tested, using simulated TX signal echoes and the SNR values set according to the values observed in field experiments. The PRS signals have turned out to be very suitable for ISAC, as they are highly configurable and enable proper adjustment between the sensing resources and communication tasks. The flexibility of the PRS configuration allows for adaptation to varying radio channel conditions, which is essential to maintain precision in sensing while preserving communication efficiency. The configurations with one PRS resource turned out to be the best choice in terms of acceptable trade-off between simultaneous efficient realization of sensing and communication tasks. They occupy two times less resources than configurations with two PRS resources while performing only slightly worse in the radar simulations. Furthermore, some changes in the PRS configuration have been proposed, which allow for a very rare distribution of PRS pilots in the 5G resource grid. It has been proven that, for good radio conditions, our suggestion provides decent results in sensing precision, whereas it does not occupy a large number of resource elements, which in turn leaves more resources for communication tasks. It also turned out that slightly irregular sampling in the time of the estimated channel frequency response, caused by interchangeable use of long and short cyclic prefixes in 5G NR waveforms and the use of some dense PRS time–frequency grid configurations, does not result in significant errors in Doppler frequency measurements. In this work, only the high-speed train scenario was simulated; however, the results obtained can be generalized and have wider impacts. This establishes a fundamental basis for further research and real-world applications in the integration of radar sensing and mobile communication.

## 7. Future Work

Based on the findings of this study, several directions for future research and development can be identified. Comprehensive field tests should be conducted to validate the simulation results in real-world environments. This would help to assess practical challenges and refine the PRS configurations for optimal performance under various conditions. Enhanced signal processing algorithms must be developed and integrated to improve the accuracy and reliability of radar sensing. This includes exploring machine learning techniques for better interpretation of the radar data. Investigating the impact of different environmental factors, such as weather conditions and urban infrastructure, on the performance of PRS-based radar sensing is essential. These future directions aim to extend the applicability and efficiency of PRS-based radar sensing, contributing to the advancement of ISAC systems in mobile networks.

## Figures and Tables

**Table 1:** 5G numerologies overview.

μ	SCS [kHz]	$N_{\mathrm{Symbols}}^{\mathrm{Slot}}$	$N_{\mathrm{Slots}}^{\mathrm{Subframe},\mu }$	$N_{\mathrm{Slots}}^{\mathrm{Frame},\mu }$
0	15	14	1	10
1	30	14	2	20
2	60	14	4	40
3	120	14	8	80
4	240	14	16	160
5	480	14	32	320
6	960	14	64	640

**Table 2:** PRS parameters for mapping to physical resources and time slots.

Parameter	Description
$l_{start}^{PRS}$	First symbol occupied by the PRS resource in a slot
$L_{PRS}$	Number of consecutive PRS symbols that are occupied in a slot
$k_{offset}^{PRS}$	RE offset in frequency domain in the first symbol occupied by the PRS resource
$K_{comb}^{PRS}$	Density of allocation of PRS pilots, e.g., for $K_{comb}^{PRS}=4$, every fourth subcarrier will be occupied by PRS pilots
$N^{RB}$	Number of RBs occupied by the PRS resource (resource bandwidth)
$I_{1st}^{PRB}$	Index of the first PRB occupied by the PRS resource
$T_{per}^{PRS}$	Periodicity in slots of a PRS resource set
$T_{offset}^{PRS}$	Offset in slots of a PRS resource set
$T_{offset,res}^{PRS}$	Offset in slots of a single PRS resource with respect to corresponding PRS resource set slot offset ($T_{offset}^{PRS}$)
$T_{rep}^{PRS}$	Number of repetitions of the single PRS resource in the single instance of the PRS resource set
$T_{gap}^{PRS}$	Slot offset between two consecutive PRS resource occurrences

## Data Availability

The original contributions presented in the study are included in the article, further inquiries can be directed to the corresponding author.

## Abbreviations

The following abbreviations are used in this manuscript:

NR	New Radio
PRS	Positioning Reference Signal
OFDM	Orthogonal Frequency Division Multiplexing
ISAC	Integrated Sensing And Communication
FFT	Fast Fourier Transform
IFFT	Inverse Fast Fourier Transform
DFT	Discrete Fourier Transform
CAF	Cross-Ambiguity Function
CIR	Channel Impulse Response
CFR	Channel Frequency Response
FDD	Frequency Division Duplex Mode
DDM	Delay–Doppler Matrix
RVM	Range–Velocity Matrix
AWGN	Additive White Gaussian Noise
SNR	Signal-to-Noise Ratio
PNFR	Peak-to-Noise-Floor Ratio
$f^{\left(D\right)}$	Doppler Frequency Shift
$x_{TX}$	Transmitted signal
$y_{RX}$	Received signal
$y_{p}$	Reflected Signal from path *p*
RE	Resource Element
RB	Resource Block
PRB	Physical Resource Block
SCS	Subcarrier Spacing
μ	Numerology
$N_{Symbols}^{Slot}$	Number of OFDM symbols per slot
$N_{Slots}^{Subframe,\mu }$	Number of slots per subframe with numerology μ
$N_{Slots}^{Frame,\mu }$	Number of slots per frame with numerology μ
$N_{cp}^{long}$	Length of the 5G NR longer cyclic prefix
$N_{pilots}^{symbol}$	Number of PRS pilots in one OFDM symbol
$N_{pilots}^{sc}$	Number of all PRS pilots on a subcarrier
